# Can we save our resources with half-body-18F-FDG-PET-CT rather than whole-body, in the management of head & neck cancers?

**DOI:** 10.1186/1470-7330-14-S1-P2

**Published:** 2014-10-09

**Authors:** LI Sonoda, A Lakhani, S Ghosh-Ray

**Affiliations:** 1 Paul Strickland Scanner Centre, Mount Vernon Hospital, London, UK

## Introduction

18F-FDG PET-CT plays a significant role in the management of head and neck (H&N) malignancies. There have been recent suggestions that half-body (above diaphragm) PET-CT may be sufficient for the management of H&N cancer patients. This study aims to determine if half-body PET-CT is a safe practice option, or should we stick to whole-body PET-CT.

## Methods

A 6-year-period retrospective analysis of 729 consecutive PET-CT scans of H&N cancer patients was performed in order to record the incidence of below-diaphragm metastases and below-diaphragm synchronous primary malignancies. The four main indications of PET-CT in H&N cancers are; pre-treatment staging of high-risk of disseminated disease, metastatic cervical lymphadenopathy with unknown primary, assessment of therapeutic response and detection of recurrence/relapse.

## Results

A total of 664 squamous cell carcinoma (SCC) and 65 nasopharyngeal carcinoma (NPC) cases were studied. 35/729 (4.8%) of cases showed below-diaphragm metastases (liver, renal, adrenal, retroperitoneal and lumbar vertebral metastases), 24/664 (3.3%) by SCC and 11/65 (16.9%) by NPC.

52/729 (7.1%) cases showed synchronous primary malignancies, of which 32 (4.4%) were below-diaphragm (colonic, pancreatic, bladder cancers and retroperitoneal lymphoma).

In total, 84/729 (11.5%) H&N cases had either below-diaphragm metastases or below-diaphragm synchronous primary malignancies.

## Conclusion

A significant proportion of H&N patients, over 10%, have either below-diaphragm metastases or below-diaphragm synchronous primary malignancies. Half-body (above diaphragm) PET-CT would have missed these lesions, leading to mis-staging of disease and mis-management of patients. It is important to keep whole-body PET-CT in practice in the management of H&N cancers. This is more so in the management of NPC compared to SCC.

